# Mechanical aberrations in hypetrophic cardiomyopathy: emerging concepts

**DOI:** 10.3389/fphys.2015.00232

**Published:** 2015-08-19

**Authors:** Dimitrios Ntelios, Georgios Tzimagiorgis, Georgios K. Efthimiadis, Haralambos Karvounis

**Affiliations:** ^1^Laboratory of Biological Chemistry, Medical School, Aristotle University of ThessalonikiThessaloniki, Greece; ^2^Department of Cardiology, AHEPA University HospitalThessaloniki, Greece

**Keywords:** hypertrophic cardiomyopathy, cardiac mechanics, stretch activation, epicardial-endocardial synergy, LVOT obstruction, myocardial disarray

## Abstract

Hypertrophic cardiomyopathy is the most common monogenic disorder in cardiology. Despite important advances in understanding disease pathogenesis, it is not clear how flaws in individual sarcomere components are responsible for the observed phenotype. The aim of this article is to provide a brief interpretative analysis of some currently proposed pathophysiological mechanisms of hypertrophic cardiomyopathy, with a special emphasis on alterations in the cardiac mechanical properties.

## Introduction

Hypertrophic cardiomyopathy (HCM) is a relatively rare inherited cardiovascular disease that affects approximately 1 in 500 young individuals and that accounts for 35% of sudden cardiac death cases in young athletes (Maron et al., 1995, 2009). It is characterized by a segmental hypertrophy of the left ventricle (LV) that mainly affects the interventricular septum, although there are no factors present that could have induced hypertrophy, such as hypertension or aortic stenosis. This type of hypertrophy has been termed asymmetrical, in contrast to the symmetrical hypertrophy in hypertensive patients, in which all LV segments are equally affected. Additional prominent features include left ventricular outflow tract (LVOT) obstruction, owing to an abnormal anterior motion of the mitral valve during systole that obstructs blood flow to the aorta, and disorganization of the myocardial tissue architecture (myocardial disarray). Some patients experience severe symptoms (dyspnea, palpitations, fainting, and sudden cardiac arrest caused by ventricular tachycardia), whereas others are asymptomatic. The onset is usually during adolescence but can occur later in life (Sherrid, 2006; Maron and Maron, 2013).

Regarding its genetic cause, myosin heavy chain beta (MYH7) and myosin binding protein C (MYBPC3) are the most commonly involved genes, followed by troponin I (TNNI3), troponin T (TNNT2), essential myosin light chain (MYL3), regulatory myosin light chain (MYL2), alpha tropomyosin (TPM1), and cardiac actin (ACTC) (Konno et al., 2010). These genes encode proteins of the sarcomere, which is the subcellular structure responsible for myocyte contraction. This paper explores how mutations in genes encoding sarcomeric proteins affect heart mechanical behavior and also focuses on unresolved issues regarding HCM disease pathomechanisms.

## The sarcomere

**Figure 1:**
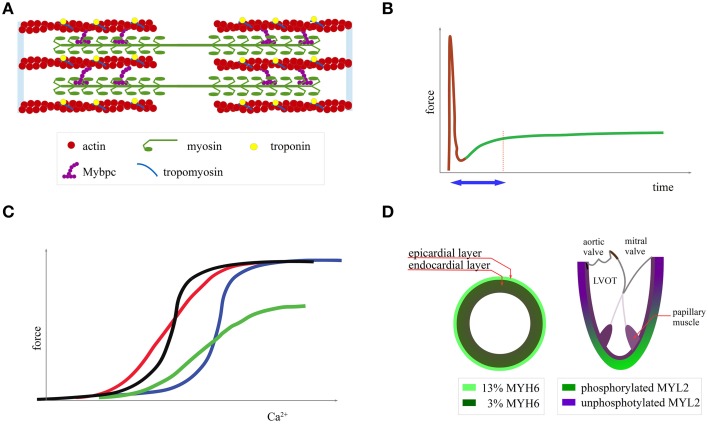
**Normal cardiac mechanics**. **(A)** Graphical representation of the sarcomere **(B)** length-dependent activation: when a myocardial fiber is subjected to stretch there is an initial passive tension (brown curve) followed, with a time delay (blue arrow), by active force development (green curve). **(C)** Ca^2+^-force curve modulation by shifting the curve along the x axis (changing myofilament affinity to Ca^2+^, black and blue curve), by changing its steepness (more graded response to Ca^2+^, red curve) and by changing maximal force (green curve) **(D)** MYH6 expression and MYL2 phosphorylation.

Sarcomeres are composed of thick filaments consisting of the molecular motor myosin and thin filaments that are formed by actin (Figure 1A). Myosin is a protein complex of two heavy chains (MYH6 or MYH7), which are the main motor, as well as two regulatory (MYL2) and two essential light chains (MYL3) with a structural and regulatory role (Harris et al., 2011). Myosin exerts force on the thin filaments by ATP hydrolysis. Because of the action of myosin, the filaments slide over each other, leading to sarcomere contraction (Fatkin and Graham, 2002). The troponins (T, I, and C) and tropomyosin form a protein complex attached to the thin filaments, which regulates contraction in response to Ca^2+^. Upon excitation and Ca^2+^ entry into the myocardial cell, Ca^2+^ binds to troponin and enables actin-myosin interaction (Fatkin and Graham, 2002). Finally, myosin binding protein C is attached to the thick and thin filaments, modulates the myosin kinetics, and is a substrate of PKA and CAMKII phosphorylation (James and Robbins, 2011). With the exception of MYBPC, where HCM mutations lead to low protein levels (frameshift mutations and deletions), a gain-of-function mutation alters the mechanical properties of these proteins (ATPase rate, force generating capacity and sliding velocity for myosin, and Ca^2+^ affinity for troponin) (Harris et al., 2011; Moore et al., 2012; Spudich, 2014). Additionally, there are normal local variations in the function of some of these proteins across different segments of the normal heart with possible relevance to the pathophysiology of HCM.

## Normal spatial patterns in the left ventricle mechanics

The advent of newer imaging technologies has favored the detailed exploration of the regional function of the heart and the quantification of myocardial deformation (myocardial strain). Myocardial deformation analysis in the form of magnetic resonance imaging (MRI) has revealed strain dissimilarities between the endocardial and epicardial layers of the left ventricle (LV) wall and among different LV segments (Bogaert and Rademakers, 2001).

This lack of uniformity in contractility is also reflected on the molecular level. More specifically, the contraction capacity of the myosin heavy chain can be either fast (MYH6) or slow (MYH7). There are certain developmental, environmental, epigenetic and hormonal factors (e.g., thyroid hormones) that regulate the expression of MYH6 and MYH7, which are encoded by a gene cluster on chromosome 14 (Gupta, 2007). MYH7 is the predominant motor in the human ventricle. MYH7 has the same force-generating capacity as MYH6 but a lower ATPase rate (reduced energy cost). Moreover, MYH6 seems to be the predominant motor in cases in which fast kinetics are required, such as in the human atrium, the contraction time of which is shorter than that of the ventricles, or in mouse ventricles, where the heart rate is as fast as 500 beats per second (Gupta, 2007). Cardiac hypertrophy and heart failure causes MYH6 downregulation (Gupta, 2007). The importance of MYH6, despite the low level of MYH6 expression in human ventricles, is also highlighted in other studies that have observed an increase in MYH6 expression in heart failure patients who improved after cardiac resynchronization therapy or medical treatment (Lowes et al., 2002; Vanderheyden et al., 2008).

Several studies have examined the mechanical properties of the epicardial and endocardial layers (Cazorla et al., 2000, 2005). For example, Stelzer et al. report that there is a transmural gradient across the porcine left ventricular wall with epicardial myocytes comprising 13% MYH6 and endocardial myocytes 3% (Figure 1D) (Stelzer et al., 2008). Another study found that the presence of MYH6, even at low levels (10%), speeds up the onset of mechanical contraction (Locher et al., 2011). Thus, subtle differences in mechanical properties exist between endocardial and epicardial layers.

In addition to the transmural heterogeneity, there is also an MYL2 phosphorylation gradient toward the apex in the murine heart (Figure 1D) (Davis et al., 2001). This post-translational modification, which increases the force and speed of contraction, favors contractility in the LV apex relating to the base (Bogaert and Rademakers, 2001; Toepfer et al., 2013). On the contrary, in the papillary muscles and the adjacent endocardial layer, there is an MYL2 hypophosphorylation (Figure 1D), leading to increased amplitude and rate of onset of the stretch-activation response (Figure 1B). In fact, the stretch activation response was shown to be critical for papillary muscle function (Vemuri et al., 1999).

## Mechanical flaws in HCM patients

The spatial patterns and control mechanisms described above are compromised in HCM patients.

### Myosin properties

Normally, a myocardial cell has two options: to express either the slow MYH7 or the fast MYH6. The HCM mutations in the MYH7 gene generally increase the myosin velocity and ATPase rate (Moore et al., 2012; Spudich, 2014). Therefore, in the presence of a dominant MYH7 mutation, the cell produces a combination of wild type slow MYH7 (normal allele) and a fast mutated MYH7. Eventually, it becomes difficult for the cell to determine the operational characteristics of the myosin that it produces. This defect may prevent the heart from utilizing certain molecular strategies to fine-tune its regional mechanical properties (e.g., transmural MYH6 gradients), thus leading to the altered pattern of ventricular contraction (ventricular dyssynchrony) that is detected in HCM patients (Chen et al., 2012). Furthermore, the increased ATPase activity in conjunction with the increased volume of the hypertrophied cell (low surface to volume ratio, increased anabolic needs) push the energy production system of the cell to its limits (Moore et al., 2012). For instance, Crilley et al. conducted a magnetic resonance spectroscopy study in which they found that even the mutation carriers that did not present any hypertrophy had a reduced phosphocreatine to ATP ratio (Crilley et al., 2003). In fact, this reduction in energy reserves was not confined to only the MYH7 mutation carriers (Witjas-Paalberends et al., 2014).

### Stretch activation response and calcium

Another important control mechanism that closely relates to the Frank-Starling law and is defective in HCM patients is length-dependent activation, which manifests as an increased force generation after myocardial stretching caused by an alteration in myofilament sensitivity to calcium (Figure 1B) (Campbell and Chandra, 2006; de Tombe et al., 2010; Sequeira et al., 2013). Myocardial cells collected from patients subjected to surgical myectomy had a defective response to stretch *in vitro* irrespective of the mutated gene (Sequeira et al., 2013). During exercise, HCM patients failed to increase stroke volume in part due to this defect (Critoph et al., 2014). An MYL3 HCM mutation in a mouse study affected the frequency range at which the heart can operate efficiently due to alteration of the stretch-activation response (Vemuri et al., 1999).

Additionally, several *in vitro* studies showed that in HCM there was a calcium ion mishandling due to reduced sarcoplasmic reticulum Ca^2+^ ATPase, alteration in Na^+^/Ca^2+^ cotransporter function, t tubule reduction, increased Ca^2+^ in the sarcoplasmic reticulum, and possibly low energy reserves, given that Ca^2+^ pumping is a process that consumes a large amount of ATP (Coppini et al., 2013; Lan et al., 2013). In addition, mutations in MYH7, MYBPC3, TNNI3, and TPM1 increased the myofilament calcium affinity and reduced the calcium-force curve steepness (Figure 1C) (i.e., a more graded response to calcium input) (van Dijk et al., 2012; Sequeira et al., 2013; Ramirez-Correa et al., 2014). A slower rate of tension development in response to a calcium influx reduces the energy efficiency because, for the same force, an increase in Ca^2+^ concentration is needed and consequently an increase in ATP consumption for Ca^2+^ recycling (Sun and Irving, 2010).

### Epicardial-endocardial synergy and disease-specific features

**Figure 2:**
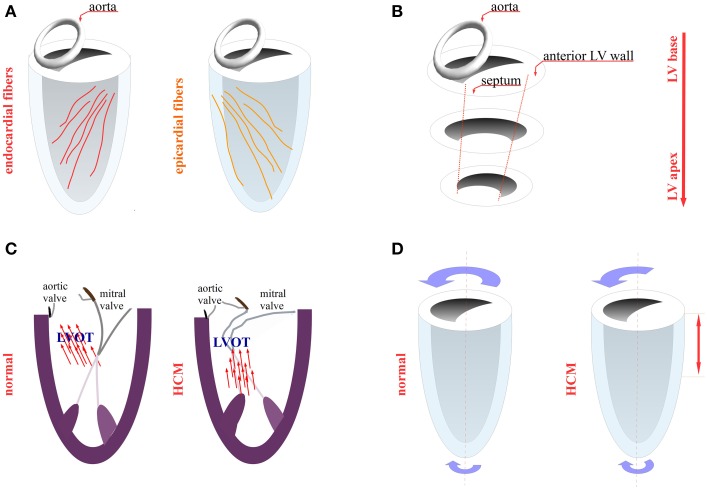
**LV mechanics in HCM**. **(A)** Endocardial fibers (red) and epicardial fibers (orange) orientation **(B)** distribution of hypertrophied areas in the left ventricle (LV) wall (spiral pattern) **(C)** left ventricular outflow tract (LVOT) obstruction due to systolic anterior motion of the mitral valve **(D)** reduced LV twist in the base and midventricular part in HCM as compared to normal subjects (red arrow).

The orientation and the sequence of activation of myocardial fibers are of paramount importance for the mechanical function of the heart. The fiber orientation across the LV wall changes from 60° to −60° from the endocardium to the epicardium (Figure 2A). During the initial phase of ventricular systole and before aortic valve opening (isovolumetric contraction), there is an activation of mainly endocardial fibers. The contraction of the endocardial fibers stretches the epicardial fibers, which is an important step for the subsequent mechanical activation of the epicardial fibers during the ejection phase (Ashikaga et al., 2009). As described above, the defect in the stretch activation response may compromise the epicardial fiber contraction, thus posing an extra mechanical load on the endocardial fibers, leading to their hypertrophy. Further evidence from an MRI study of the hypertrophy distribution shows that that hypertrophy typically starts in the anteroseptal region and spirals in a counterclockwise manner toward the apex (Figure 2B) (Florian et al., 2012). According to the Torrent Guasp myocardial band model, the hypertrophied segment corresponds to the so-called descending helix (endocardial fibers) (Buckberg et al., 2008; Sengupta and Narula, 2012). Likewise, a myocardial deformation analysis, in which MRI was used, revealed that the endocardial fibers were contracting properly, whereas the epicardium was hypocontractile with non-contracting or even stretched segments (Aletras et al., 2011). Whether the hypokinesis of the epicardial fibers in patients may facilitate the emergence of myocardial disarray in this area, much like the disarray observed in detached papillary muscles after mitral valve replacement surgery, needs to be explored (Pirolo et al., 1982). Indeed, a human MRI study showed myocardial disarray in hypokinetic areas (Tseng et al., 2006). Conversely, in an HCM mouse model (MyBPC knockout), the MRI revealed an increased disarray in the endocardial side, although this finding was not histologically confirmed (Wang et al., 2010).

There is increasing evidence that this abnormal coordination between the epicardial and the endocardial fibers may relate to LVOT obstruction. The heart orchestrates the contraction of different segments to achieve decreased flow turbulence and handle energy losses (Sengupta et al., 2012). Furthermore, the coordinated movements of the epicardial and endocardial fibers during isovolumetric contraction and relaxation are crucial for the proper flow redirection between the outflow and the inflow tract (Sengupta et al., 2007). For example, when alterations occur in the heart contraction pattern, because of the epicardial pacing from the left ventricle base, there are also observable effects on the intracavitary flow pattern (Goetz et al., 2005; Sengupta et al., 2007). In patients with LVOT obstruction, there was a misdirection of flow toward the outflow tract during ejection. In particular, the flow affected the posterior aspect of the mitral valve, causing its anterior displacement, thus obstructing the outflow tract (Figure 2C) (Ro et al., 2014). In HCM, the left ventricle adopted an abnormal contraction pattern, characterized by an increased deformation in the circumferential direction and a decreased deformation in the longitudinal (base to apex) direction. Additionally, the increased circumferential strain in obstructive vs. non-obstructive HCM may contribute to LVOT obstruction (Carasso et al., 2008; Ntelios et al., 2015). Concerning the alleviation of the LVOT obstruction in some patients after implantation of a pacemaker, pacing from the right ventricle apex may alter the LV contraction pattern and redirect flow in an advantageous way (Sherrid, 2006). Another factor that facilitates LVOT obstruction is the elongation of the mitral valve leaflets (Ro et al., 2014). This defect may be secondary to the abnormal intracavitary flow pattern because the hydrodynamic conditions are of paramount importance for proper valve formation during the developmental stages (Kalogirou et al., 2014).

Owing to the geometrical configuration of the epicardial and endocardial fibers, during systole, the left ventricle performs a wringing motion (Figure 2D). This motion is quantitatively assessed as a “ventricular twist” (the difference of rotation between the base and the apex). The physiological role of this ventricular twist is to homogenize the wall stress across the ventricular wall, thus increasing the involvement of the epicardial fibers during contraction (Sengupta et al., 2008). Carasso et al. observed that in HCM patients the net ventricular twist did not differ from the control group (Carasso et al., 2008). Nonetheless, the rotation of the midventricular part, relating to the left ventricle base, was minimal, and the apical part was the main contributor to the total angle of rotation (Figure 2D) (Carasso et al., 2008). Therefore, this locally reduced twist in the base and midventricular part may be an additional factor increasing the mechanical load of the endocardial fibers, leading to their hypertrophy. In fact, HCM patients failed to increase the ventricular twist during exercise (Soullier et al., 2012). Another important consideration is that the ventricular twist depends on geometrical factors, such as the ventricular length and diameter (Young and Cowan, 2012), which can be modified in a hypertrophied heart.

### Apical hypertrophy

In some cases, hypertrophy predominately involves the LV apex (Chen et al., 2011). Some studies have shown that HCM patients present a blunted base to apex gradient in contractility and have reduced apical rotation and stretched apical segments (Reddy et al., 2008; Chang et al., 2010). These characteristics highlight the reduced systolic function of the apex in these patients. Theoretically, defects in the MYL2 and other unrecognized mechanisms that enhance apical contraction might lead to compensatory apical hypertrophy. Additionally, aneurysm formation is an anticipated complication given the increased apical wall stress (Guccione et al., 1995; Chen et al., 2011). Furthermore, mutations in the MYL2 and MYL3 genes are connected to the midcavitary hypertrophy variant (Poetter et al., 1996). In this HCM subtype, there is hypertrophy of the midventricular part of the LV wall and papillary muscles possibly because MYL3 and MYL2 can modulate the amplitude and rate of onset of the stretch-activation response, thus compromising papillary muscle function (Poetter et al., 1996; Vemuri et al., 1999; Arad et al., 2005; Stelzer et al., 2006). Another important clinical observation is that patients with the same mutation present an apical or a more classical asymmetrical hypertrophy pattern (Arad et al., 2005). Additionally, 10% of HCM patients present apical hypertrophy with concurrent septal hypertrophy (Florian et al., 2012). Thus, the different phenotypes in these patients may simply reflect the variable extent of hypertrophy toward the base.

## Treatment options in HCM

The treatment of HCM has so far largely focused on treating the symptoms of the disease using beta adrenergic receptor blockers. B-blockers slow the heart rate and reduce myocardial oxygen consumption and LVOT obstruction. Disopyramide and the calcium channel blocker verapamil are also helpful. In cases of drug-refractory symptoms, alleviation of outflow tract obstruction by either surgical septal myectomy or percutaneous infusion of alcohol in the septal branch of the left coronary artery is the current practice. A drug-based approach targeting length-dependent activation, myofilament Ca^2+^ affinity and myosin properties is very promising. Another important issue in the management of these patients is risk stratification for sudden cardiac death (SCD). In general terms, patients with unexplained syncope, documented ventricular tachycardia or aborted SCD, positive family history for SCD, maximal LV wall thickness >30 mm and abnormal drop in blood pressure during exercise, treated with an implantable defibrillator (Maron and Maron, 2013; Efthimiadis et al., 2014).

## Conclusion

Hypertrophic cardiomyopathy exhibits significant heterogeneity (i.e., numerous mutations and a variable clinical course). The mechanisms analyzed in this paper shed some light on the relation of mutations to disease phenotype, although they may not necessarily apply to all patients. A better understanding of the pathophysiological mechanisms leading to hypertrophy will contribute to the further improvement of treatment and to risk stratification for sudden cardiac death.

### Conflict of interest statement

The authors declare that the research was conducted in the absence of any commercial or financial relationships that could be construed as a potential conflict of interest.
